# Matcha green tea beverage moderates fatigue and supports resistance training-induced adaptation

**DOI:** 10.1186/s12937-023-00859-4

**Published:** 2023-07-05

**Authors:** Mizuho Shigeta, Wataru Aoi, Chiharu Morita, Kurumi Soga, Ryo Inoue, Yoichi Fukushima, Yukiko Kobayashi, Masashi Kuwahata

**Affiliations:** 1 Laboratory of Nutrition Science, Division of Applied Life Sciences, Graduate School of Life and Environmental Sciences, Kyoto Prefectural University, 1-5 Hangi-Cho Shimogamo, Sakyo-Ku, Kyoto, 606-8522 Japan; 2 Laboratory of Animal Science, Department of Applied Biological Sciences, Faculty of Agriculture, Setsunan University, Osaka, Japan; 3 Department of Health Science, Faculty of Sports and Health Science, Daito Bunka University, Saitama, Japan

**Keywords:** Muscle adaptation, Resistance training, Matcha green tea, Fatigue, Gut microbiota

## Abstract

**Background:**

Resistance training adaptively increases muscle strength and mass, contributing to athletic performance and health promotion. Dietary intervention with natural foods provides nutrients that help accelerate muscle adaptation to training. Matcha green tea contains several bioactive factors such as antioxidants, amino acids, and dietary fibers; however, its effect on muscle adaptation is unclear. In this study, we aimed to investigate the effects of matcha beverage intake on muscle adaptation to resistance training.

**Methods:**

Healthy, untrained men were randomized into placebo and matcha groups. Participants consumed either a matcha beverage containing 1.5 g of matcha green tea powder or a placebo beverage twice a day and engaged in resistance training programs for 8 (trial 1) or 12 weeks (trial 2).

**Results:**

In trial 1, maximum leg strength after training tended to increase more in the matcha group than that in the placebo group. In the matcha group, subjective fatigue after exercise at 1 week of training was lower than that in the placebo group. Gut microbe analysis showed that the abundance of five genera changed after matcha intake. The change in *Ruminococcus*, *Butyricimonas*, and *Oscillospira* compositions positively correlated with the change in maximum strength. In trial 2, the change in skeletal muscle mass in response to training was larger in the matcha group. In addition, the salivary cortisol level was lower in the matcha group than that in the placebo group.

**Conclusion:**

Daily intake of matcha green tea beverages may help in muscle adaptation to training, with modulations in stress and fatigue responses and microbiota composition.

**Supplementary Information:**

The online version contains supplementary material available at 10.1186/s12937-023-00859-4.

## Background

Skeletal muscles support physical activity and act as a major metabolic organ. In athletics, increased muscle strength and mass are important factors that improve performance. Age-related muscle loss and decreased strength, referred to as sarcopenia, have been recognized as major risk factors and may necessitate nursing care in aged individuals. As a counter-intervention, resistance exercise training has been recommended to improve athletic performance and prevent sarcopenia in older adults [1]. Resistance training increases the strength of skeletal muscles and causes hypertrophy as typical adaptation effects. In the early stage of training, the participation of muscle fiber in muscle contraction is increased to support lifting weights. Subsequently, continuous resistance training causes muscle fiber hypertrophy with elevated protein anabolism, resulting in further improved muscle strength [2]. Muscle mass also contributes to whole-body energy expenditure and substrate utilization, which is associated with the development of obesity and metabolic diseases.

Adequate dietary nutrition is necessary for muscle adaptation to training. Sufficient protein intake is required to supply amino acids as substrates for protein synthesis. The timing of protein intake and complementary consumption of carbohydrates effectively activate protein anabolism [3]. Moreover, adequate nutrition management prevents fatigue and promotes supercompensation, which can have beneficial effects on adaptation. In addition, some micro-compounds contained in natural foods have been shown to regulate protein metabolism and suppress physical fatigue under in vivo and in vitro experimental conditions. Amino acids act as substrates of constitutive proteins and signaling factors of accelerated protein synthesis [4]. Some factors with antioxidative potential modulate oxidative stress, which can inhibit muscle contraction and cause mitochondrial dysfunction [5, 6]. However, intervention studies on the role of dietary foods in muscle adaptation to resistance training in humans have been limited.

Tea beverages, which are consumed daily, are sources of bioactive micro-compounds. Green tea contains high concentrations of catechins and reportedly has various health benefits. Daily intake of green tea is beneficial for neural, cardiovascular, and metabolic functions in humans [7-9]. The combination of green tea intake and exercise training accelerates aerobic metabolism and lipid utilization in skeletal muscle [10]. Matcha—a powdered green tea—contains specific functional compounds. It is prepared from tea leaves harvested after growing plants under shaded conditions for several weeks. After processing, the final product is consumed as a thick suspension; its catechin content per cup is twice that of normal (sencha) green tea infusions [11]. Matcha green tea powder contains carotenoids, including lutein, theanine, vitamin K, and dietary fibers, which are found in relatively low levels in sencha green tea [12]. Catechins and carotenoids have antioxidant and reactive oxygen species (ROS)-scavenging effects. During exercise, the antioxidant activity can suppress excess oxidative stress in muscle cells, endothelial cells, and neutrophils. Dietary fibers improve the intestinal environment by modulating the microbiota profile and epithelial barrier function and regulate nutrient absorption [13, 14]. Catechins may also efficiently modulate the intestinal environment through antibiotic and antioxidant effects [15]. A favorable intestinal environment can improve metabolic function and ameliorate fatigue and psychological stress [16, 17]. The amino acid theanine reduces stress and elevates nutrient metabolism by affecting the central and peripheral nervous systems [18, 19]. Therefore, the micro-compounds contained in matcha may promote recovery and protein synthesis, resulting in muscle adaptation. In this study, we aimed to investigate the effect of the daily consumption of matcha on resistance training-induced adaptation in humans.

## Methods

### Participants

Thirty-six young and healthy men participated in this study, which was approved by the ethics committee of Kyoto Prefectural University (No. 2017–146). All participants provided written informed consent. None of the participants suffered from current (at the time of the study) or prior chronic diseases or had a history of smoking. Furthermore, none of the participants were using any medication or supplements at the time of the study or habituated to regular exercise. The participants were randomly divided into placebo and matcha groups, and body composition parameters—body weight, body fat, muscle mass, and body mass index (BMI)—were measured using bioelectrical impedance analysis (InBody430; InBody Co., Ltd., Seoul, Korea).

### Experimental design

**Fig. 1:**
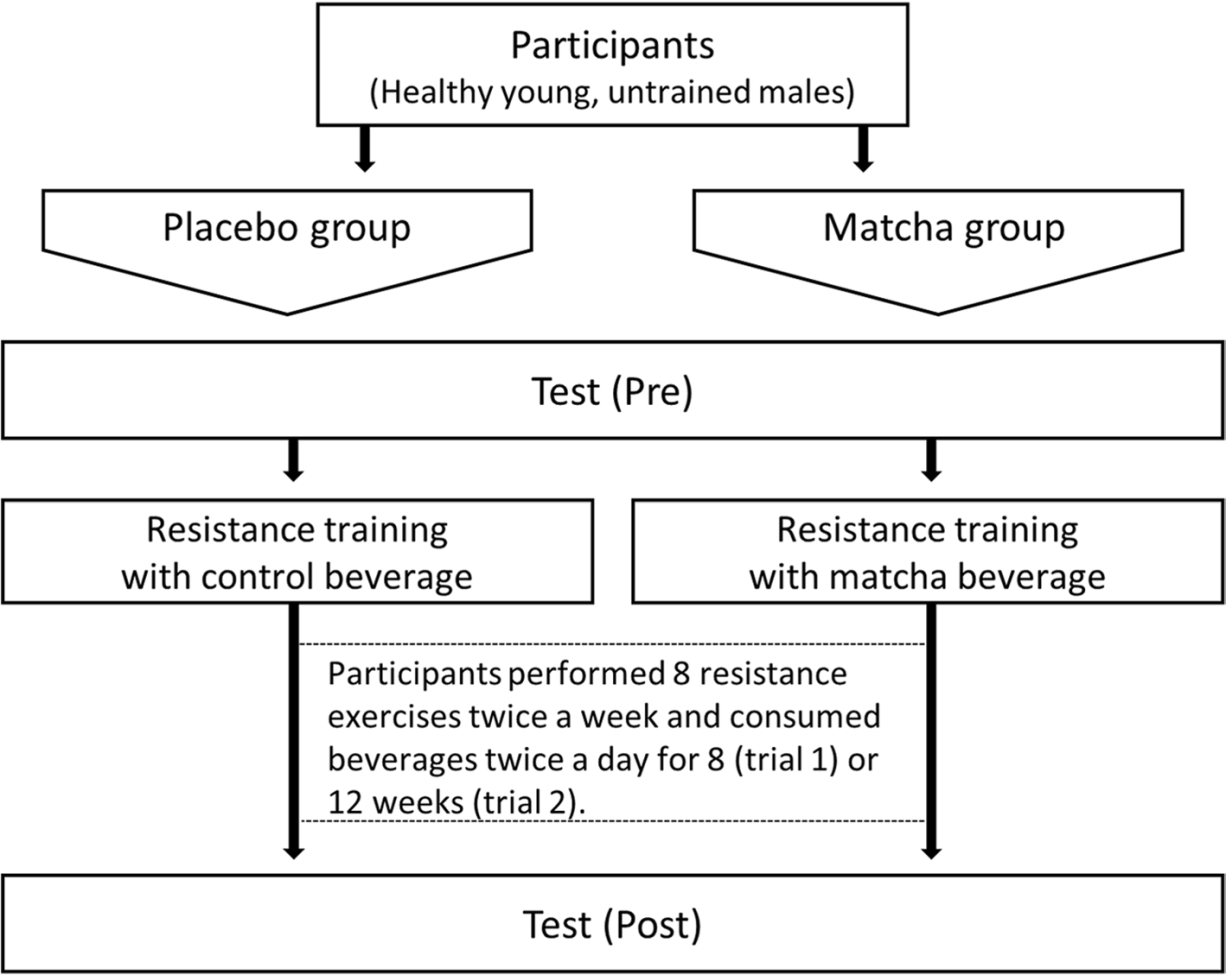
Experiment protocol

This study involved two randomized placebo-controlled trials (Fig. 1). In trial 1, 17 participants (age: 22.3 ± 0.3 years) were randomly divided into placebo (*n* = 8) and matcha (*n* = 9) groups and instructed to follow a resistance training program twice a week for 8 weeks. Body composition, maximum muscle strength, whole-body energy expenditure, and blood parameters were measured during the week before commencing the training period and the final week of training (Figure S1). The level of subjective fatigue was measured on the first exercise day and on an exercise day during the final week of training. Fecal samples were collected before and after 4 and 8 weeks of intervention. In trial 2, 19 participants (age: 20.9 ± 1.3 years) were randomly assigned to placebo (*n* = 10) and matcha (*n* = 9) groups and instructed to engage in a resistance training program twice a week for 12 weeks. Body composition, saliva parameters, and visual function were measured during the week before and after the intervention period.

During the trial period, the participants of both groups consumed beverages twice a day. The matcha group consumed a beverage containing 1.5 g matcha green tea powder [contents/cup; 0.06 g water, 0.4 g protein, 0.2 g fat, 4.4 g carbohydrate, 166.5 mg total polyphenols, 94.5 mg epigallocatechin gallate (EGCG), 1.1 mg lutein, 46.4 mg vitamin K, 27 mg theanine, 488 mg dietary fiber, and 45 mg caffeine] (Nestlé Japan Ltd., Kobe, Japan). The placebo group consumed a placebo tea-flavored beverage (contents/cup; 0.06 g water, 0 g protein, 0.1 g fat, 4.4 g carbohydrate, and 45 mg caffeine) daily throughout the trial period.

### Resistance exercise training

The training program consisted of eight resistance exercises: chest press, fly, back extension, seated rowing, leg press, leg extension, leg curl, and sit-up, performed using a combined exercise machine (Senoh Co., Ltd., Chiba, Japan). The participants performed 3 sets of 10 repetitions at a 10-repetition maximum (RM). Training frequency was twice a week at 2–3-day intervals, and weight load was gradually increased according to the 10 RM of individuals. Maximum leg extension strength was measured in both legs using a knee-extension strength meter (ST 200R; Meiko Co., Ltd., Osaka, Japan). The grip strength of both hands was assessed using GRIP D (T.K.K. 5401; Takei Scientific Instruments Co., Ltd., Osaka, Japan).

### Indirect metabolic performance

The participants were instructed to refrain from intense physical activities, eating, and drinking, except for water, from 22:00 until breakfast in the morning. In addition, they were requested to eat 200 g of steamed rice (energy, 312 kcal; protein, 5.0 g; fat, 0.6 g; and carbohydrate, 74.2 g) without caffeine and alcohol 1 h before visiting the laboratory. After sitting for 30 min, the oxygen consumption and carbon dioxide production levels of the participants were measured in the supine position using a breath-by-breath respiromonitor system (AE-310 s; Minato Medical Sciences Co., Ltd., Osaka, Japan) for 15 min. Respiratory quotient and substrate use (carbohydrate and fat oxidation) were calculated from the levels of oxygen consumption and carbon dioxide production, as described previously [20].

### Blood parameters

The participants were instructed to refrain from intense physical activity and fast from 22:00 h on the day before blood sample collection. On the day of blood sampling, each participant ate 200 g of steamed rice and rested for 1 h. Blood samples were collected before and after resistance exercise (8 exercises, 3 sets of 10 repetitions at 10 RM, as mentioned above) during the pre- and post-intervention periods. The collected blood was injected into a vacuum blood collection tube and centrifuged at 1,800 × *g* at 4 °C for 15 min. The obtained serum samples were used to measure carbonylated protein concentration and creatine kinase activity using enzyme-linked immunosorbent assay (ELISA) kits (BioCell Co., Ltd., Auckland, New Zealand; BioAssay Systems, Hayward, CA, USA) in accordance with the manufacturer's instructions.

### Subjective fatigue

The degree of subjective fatigue before exercise (at rest) was measured using a visual analog scale. The participants were asked to indicate their degree of subjective fatigue on a 100-mm horizontal line, with the left end (0 mm) indicating “no fatigue” and the right end (100 mm) indicating “maximum fatigue.”

### Analysis of fecal microbiota

Brushes and sheets for stool collection were distributed to the participants, and stool samples were collected before, at week 4, and at week 8 of the intervention. Stool samples were refrigerated, and bacterial DNA was extracted within 3 weeks after collection. Metagenomic analyses of 16S rRNA of the extracted DNA samples were performed using a next-generation sequencer (MiSeq; Illumina K.K., Tokyo, Japan). Bacterial DNA extraction from feces, library preparation, and deep sequencing were performed as previously described [21]. Sequence data were analyzed as previously described [22] using QIIME2 version 2018.8.

### Saliva parameters

To avoid the effect of the circadian rhythm, saliva was collected at the same time of day before and after the intervention, using a saliva collection kit (Salivette, Sarstedt, Germany) consisting of a centrifuge tube and sterile cotton. After rinsing the mouth with distilled water for 30 s, each participant, wearing rubber gloves, placed a sterile cotton swab in their mouth and chewed for 1 min at a mastication rate of 1 chew/s. The cotton swab, which absorbed saliva secreted in response to the chewing stimulus, was centrifuged (4 °C, 1,800 × *g*, 15 min) to collect the saliva, which was stored at − 80 °C until use. Salivary cortisol and secretory IgA (sIgA) concentrations were measured using ELISA kits (Salimetrics, Trier, Germany), and their amounts were calculated from saliva concentration and volume.

### Visual function

Two methods were used to evaluate the participants' ability to visually discern a moving object. Forward and backward kinetic visual acuity (KVA) was measured using a dynamic vision meter (AS-4; Kowa Co., Ltd., Tokyo, Japan). Lateral dynamic visual acuity (DVA) and ocular motor skills (OMS) were evaluated on a computer monitor using sports vision software (ArrowZeye; Diamond Eye Co., Ltd., Tokyo, Japan) [23]. The participants were subjected to DVA and OMS tests at a distance of 60 cm from a 34-cm monitor, and the percentage of correct answers was evaluated. In the DVA test, the participants identified numbers moving from left to right across the screen. In the OMS test, nine locations on the screen randomly flashed three circles and six squares. The participants were required to recognize the three circles and indicate their locations.

### Dietary assessment

A dietary assessment was conducted to calculate nutrient intake before trial commencement. All participants were permitted to eat freely, and their food intake was recorded for 3 days using a food diary and camera. Thereafter, a dietitian reviewed the recorded data to follow up and estimate participants' nutrient intake using Excel add-in software (Excel Eiyou-kun Ver. 6.0; Kenpakusha Co., Ltd., Tokyo, Japan).

### Statistical analyses

All data are reported as mean ± standard deviation. A two-way analysis of variance (ANOVA) was conducted to assess the significance of the interaction between drink intervention (group) and time. Post hoc analyses were conducted using Bonferroni’s test to compare significant interactions following ANOVA. An intra-group comparison was conducted if the main effect of time without interaction was observed. Differences in changes between the placebo and matcha groups were evaluated using the Mann–Whitney U test or an independent samples *t*-test, depending on whether they were normally distributed. Spearman's rank-order correlation coefficient was used to estimate bivariate correlations. Statistical analysis was performed using SPSS Statistics for Windows, Version 25.0 (SPSS Inc., Chicago, IL, USA). Statistical significance was set with two-sided *P-*values < 0.05 and a trend at < 0.1.

## Results

### Body composition and muscle strength during the 8-week intervention

In trial 1, no significant interactions and changes in response to training in body weight, BMI, skeletal muscle mass, or body fat percentage were observed (Table 1). In the placebo and matcha groups, training significantly increased the maximum strength for leg and chest presses (*P* < 0.01); however, no significant interactions were observed for both chest and leg presses (*P* < 0.1) (Table 2). The change in strength for leg presses tended to be higher in the matcha group than that in the placebo group (*P* < 0.1). Knee-extension strength showed a significant increase due to training in the dominant leg (*P* < 0.05) but not in the non-dominant leg in the placebo group (*P* < 0.1) and in both dominant (*P* < 0.05) and non-dominant (*P* < 0.05) legs in the matcha group, but not significant interaction. No between-group differences concerning habitual intake of total energy, protein, fat, and carbohydrate were observed (Table S1).

**Table 1:**
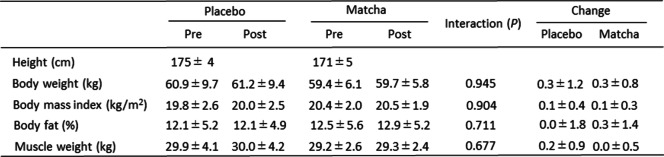
Physical characteristics of the participants in trial 1

Values are expressed as mean ± standard deviation. Pre: pre-training, Post: post-training

**Table 2:**
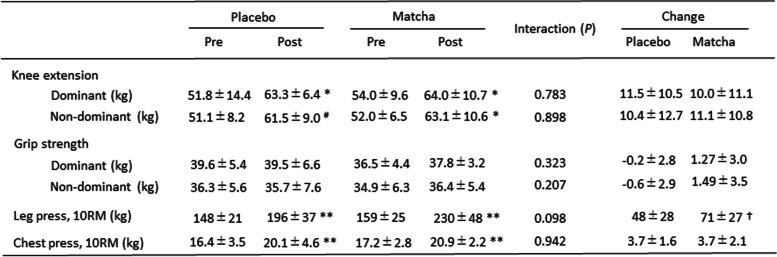
Muscle strength in trial 1

Values are expressed as mean ± standard deviation

^#^*P* < 0.1, *P < 0.05, ** *P* < 0.01 vs. Pre. †* P* < 0.1 vs. Placebo. Pre: pre-training, Post: post-training

### Blood and fatigue parameters during the 8-week intervention

**Fig. 2:**
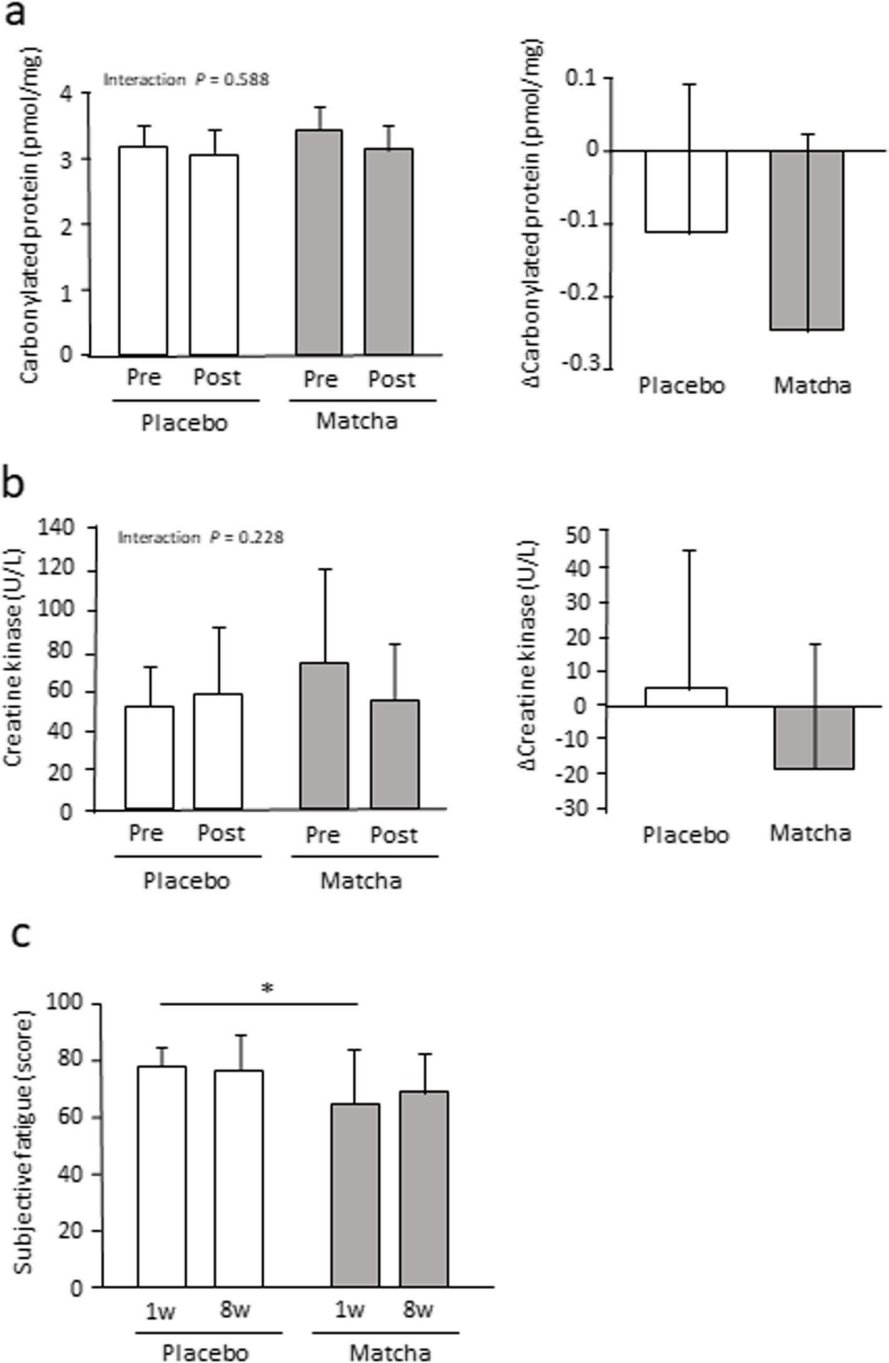
Blood and subjective fatigue parameters in trial 1. Serum carbonylated protein concentration (**a**) and creatine kinase activity (**b**) before and after the intervention. Subjective fatigue levels (**c**) during the intervention at weeks 1 and 8. * P < 0.05. Values are expressed as mean ± standard deviation. Pre: pre-training, Post: post-training

The concentration of serum carbonylated proteins and creatine kinase activity neither showed significant interactions nor training-induced changes (Fig. 2a, b). Carbonylated proteins tended to be decreased by training in the matcha group (*P* < 0.1). Subjective fatigue levels significantly differed between the groups, being lower in the matcha group than those in the placebo group at week 1 (*P* < 0.05) (Fig. 2c).

No differences in resting oxygen consumption, respiratory quotient, carbohydrate oxidation, and fat oxidation were observed between the groups before and after the intervention (Table S2).

### Gut microbiota during the 8-week intervention

**Fig. 3:**
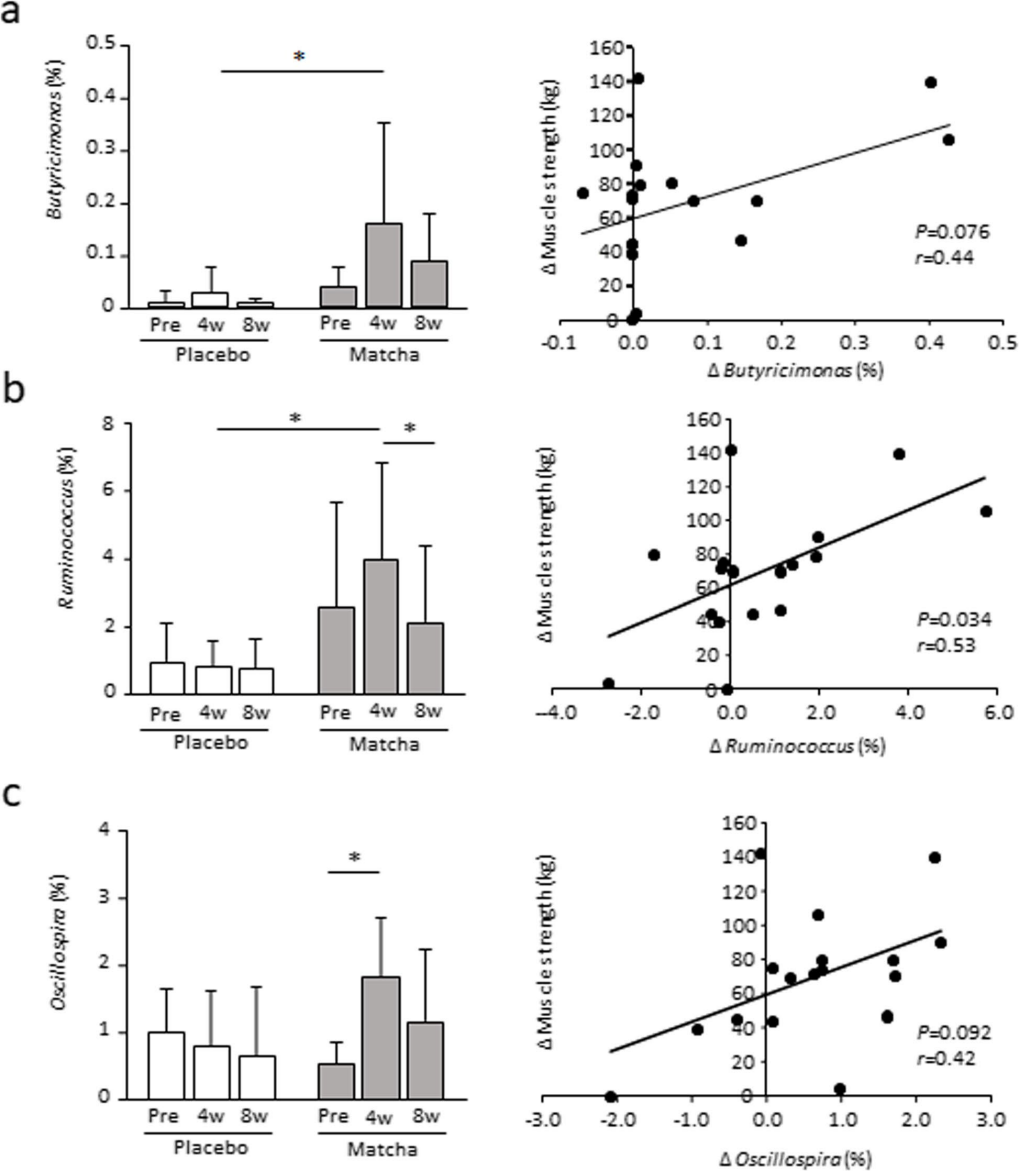
Abundance of gut microbiota genera in trial 1. Proportion of the genera *Butyricimonas* (**a**), *Ruminococcus* (**b**), and *Oscillospira* (**c**) before and at weeks 4 and 8 during the intervention. Correlation analyses between the change in maximum strength for the leg press and the change in genera percentage at week 4 compared with pre-intervention levels are shown in the right panel. * P < 0.05. Values are expressed as mean ± standard deviation. Pre: pre-training

The Chao1 and Shannon indices, which indicate the alpha diversity of gut bacteria, were not altered by the intervention within or between groups. However, the abundance of five genera changed significantly after the intervention. The abundance of *Oscillospira* and *Ruminococcus* in the matcha group at week 4 was significantly higher than that before the intervention or at week 8 (*P* < 0.05) (Fig. 3, Table S3). The abundance of *Ruminococcus*, an unclassified genus belonging to the family *Ruminococcaceae*, and *Butyricimonas* was significantly higher, and that of *Dorea* was significantly lower at week 4 in the matcha group than in the placebo group (*P* < 0.05). The change in maximum strength for the leg press showed a significant positive correlation with the change in abundance of the genera *Ruminococcus* at week 4 compared with that before intervention (*P* < 0.05) and tended to be positively correlated with *Butyricimonas* and *Oscillospira* abundance (*P* < 0.1) (Fig. 3).

### Body composition, stress-related parameters, and visual function during the 12-week intervention

**Fig. 4:**
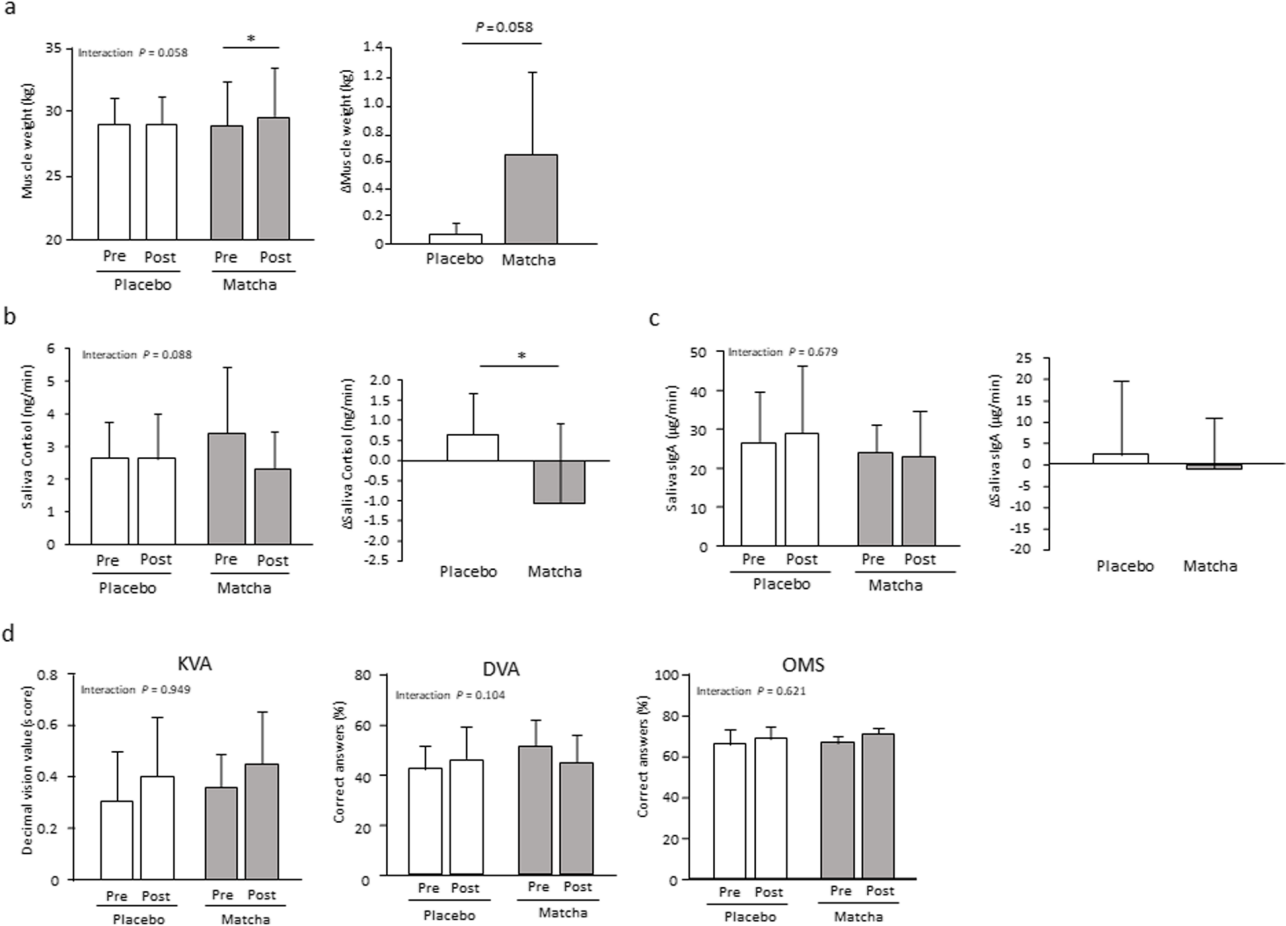
Muscular, stress, and visual parameters in trial 2. Muscle weight (**a**), salivary cortisol (**b**), salivary sIgA (**c**), and visual function (**d**) before and after the intervention. *P < 0.05. Values are expressed as mean ± standard deviation. Pre: pre-training, Post: post-training, KVA: kinetic visual acuity, DVA: dynamic visual acuity, OMS: ocular motor skills

Generally, the adaptation of skeletal muscles to resistance training results in an initial increase in muscle strength, followed by muscle hypertrophy with an extended training period. Therefore, we conducted trial 2 with an intervention period of 12 weeks. Stress-related parameters in saliva and visual ability were also examined because the outcomes of trial 1 and previous studies suggested that matcha may suppress the stress response [24, 25]. Skeletal muscle mass showed a significant increase in response to training for 12 weeks in the matcha group (*P* < 0.05) with a trend indicating interaction (*P* < 0.1), with the change being larger in the matcha group than that in the placebo group (*P* < 0.1) (Fig. 4a). Body weight, BMI, and fat percentage did not show any significant interactions; the change in fat percentage was significantly lower in the matcha group (*P* < 0.05) (Table S4). No significant differences in habitual dietary energy, protein, fat, and carbohydrate intake were observed between the groups (Table S5).

Among stress-related parameters, the salivary cortisol secretion rate showed a trend indicating interaction (*P* < 0.1) (Fig. 4b). The change in secretion rate was significantly lower in the matcha group than that in the placebo group (*P* < 0.05). No significant interaction and changes between groups were observed in the rate of salivary sIgA secretion (Fig. 4c). Regarding visual function, there were no significant interactions in KVA, DVA, and OMS (Fig. 4d). OMS tended to be increased by training in the matcha group (*P* < 0.1).

## Discussion

In this study, we investigated the effects of matcha green tea consumption on adaptation to resistance training in young men. After the 8-week intervention (trial 1), a greater change in leg strength was found in the matcha group. Furthermore, a higher muscle weight in response to training for 12 weeks was found in the matcha group (trial 2). These results suggest that dietary matcha green tea may accelerate muscle adaptation to resistance training. During adaptation to resistance exercise training, more muscle fibers are mobilized in the early stage. Muscle fibers become thicker with continued training; therefore, an extended training period results in significant muscle mass gain, further increasing muscle strength [2]. Lower fatigue and stress responses might promote recovery, leading to rapid adaptation and, ultimately, increased muscle strength and mass.

The change in salivary cortisol secretion was lower in the matcha group than that in the placebo group. This salivary indicator indirectly reflects the activation of the sympathetic nervous and hypothalamic-adrenal systems associated with stress [26]. Daily administration of matcha reduces anxiety-like behaviors in response to psychological and physiological stresses in mice [25]. Theanine, a typical ingredient of matcha, may prevent stress. Theanine in circulation crosses the blood–brain-barrier and modulates neural transmission in the brain by reducing the activation of post-synaptic glutamate receptors through the inhibitory function of glutamine in neurons [19]. Theanine consumption decreases stress and fatigue responses, including salivary cortisol and subjective stress levels in humans [19]. Although the amount of theanine contained in two cups of matcha green tea is less than the effective amount, the combined intake of theanine and other ingredients might be beneficial.

Oxidative stress may cause fatigue owing to excessive exercise [27]. ROS produced during exercise cause the oxidation of lipids and proteins in cells, resulting in oxidative damage. Oxidative stress is easily elevated in individuals without exercising habits because of their low antioxidant capacity [28]. The sarcoplasmic reticulum proteins, such as ryanodine receptor and calcium-dependent ATPase, are easily oxidized and inactivated by ROS [29]. This leads to continuously higher cytosolic calcium levels, which prevents continuous muscle contraction and leading muscle fatigue. Additionally, ROS activate inflammatory mediators that cause delayed-onset muscle damage [30]. Two cups of matcha green tea powder (3.0 g) as a thick suspension provide approximately 300 mg of catechin, twice the amount provided by sencha green tea. A major catechin, EGCG, can exert antioxidant and anti-fatigue effects [31]. In addition, matcha contains several other antioxidants, such as lutein, that scavenge ROS and suppress inflammatory responses. One cup of matcha green tea contains an equivalent amount of lutein consumed daily by Japanese individuals [32]. Although the concentration of serum carbonylated proteins, a product of oxidatively modified proteins, did not vary between groups in the present study, the irrelevance of oxidative stress in skeletal muscle fatigue could not be ascertained. In addition, since variations in exercise-induced ROS and target organelles are expected, the measurement of various parameters is required to examine the modulative effect of redox status in skeletal muscle.

Visual function is also associated with physical fatigue and has been shown to be lower in fatigued individuals than in their non-fatigued counterparts [33, 34]. A recent study showed that visual motion processing for optokinetic responses was enhanced in mice administered green tea or a green tea catechin, EGCG [35]. In addition, lutein, a specific compound in matcha green tea, is transported from circulation into the neural retina and accumulates in the macula lutea of the human eye. Thus, it is involved in an antioxidative capacity and protects visual function [36]. Although matcha is potentially beneficial to visual function, in the present study, it did not show significantly affect the related parameters in participants who consumed matcha under training conditions. Therefore, further studies are required to examine its effect on visual function. Given that antioxidants can support visual function, they may be more beneficial under conditions of high oxidative stress, for example, in older people and elite athletes, than in young, healthy subjects.

Metagenomic analysis of gut microbiota revealed significant changes in five genera of bacteria following matcha intake. *Butyricimonas* maintain immune and metabolic functions by producing butyric acid [37]. *Ruminococcus* and *Oscillospira* enhance dietary fiber metabolism and produce short-chain fatty acids that act as metabolic regulators in the host [38, 39]. Dietary fibers and antioxidants in matcha can improve the intestinal environment. Changes in the abundance of *Butyricimonas*, *Ruminococcus*, and *Oscillospira* positively correlated with increased maximum muscle strength. Although the causal relationship between the changes is unclear, an improved microbiota profile may lead to stress-response hormone suppression, parasympathetic dominance, and decreased insomnia and anxiety test scores [40]. In contrast, dysbiosis causes impaired intestinal permeability, accelerating invasion by endotoxins and antigens into the blood stream and disturbing metabolic and immune functions throughout the body [41]. Therefore, an improved intestinal environment may be involved in metabolic function and fatigue in skeletal muscles, further strengthening adaptation. The notable change in gut microbiota found after 4 weeks of intervention was not observed after 8 weeks. The composition of the human gut microbiota is generally determined during childhood and is thought to return to its original state even after undergoing transient changes due to environmental factors, such as altered dietary habits [42]. In addition, matcha intake combined with exercise training might be a critical condition required to realize these changes. Future studies are required to examine the changes in gut microbiota induced by matcha consumption and the functional effects of these changes.

## Conclusions

Collectively, matcha green tea consumption during resistance training modulates muscle adaptation. Compounds in matcha can moderate exercise-induced stress and fatigue responses, which may promote recovery after exercise and training-induced adaptation. In addition, positive correlations were found between changes in muscle adaptation and microbiota. Further studies should examine the detailed mechanism of action of matcha and the significance of microbiota modulation. Although the amounts of some compounds in matcha are less than the effective amount, beneficial effects may still be obtained as the compounds are taken in combination.

## Supplementary Information

**Additional file 1: Figure S1.** Time-course of assessment.

**Additional file 2: Table S1.** Dietary intake of energy and nutrients in trial 1.

**Additional file 3: Table S2.** Energy expenditure in trial 1.

**Additional file 4: Table S3.** Proportion of microbiota genera in trial 1.

**Additional file 5: Table S4.** Physical characteristics of participants in trial 2.

**Additional file 6: Table S5.** Dietary intake of energy and nutrients in trial 2.

## Data Availability

The datasets used and/or analyzed in this study are available from the corresponding author upon reasonable request.

## Abbreviations

BMI: Body mass index
RM: Repetition maximum
KVA: Kinetic visual acuity
DVA: Dynamic visual acuity
OMS: Ocular motor skill
ROS: Reactive oxygen species
