# Supplementary figures and images for: Matcha green tea beverage moderates fatigue and supports resistance training-induced adaptation
Source: Nutr J. 2023 Jul 5;22:32. doi: 10.1186/s12937-023-00859-4 (PMC10320999; doi:10.1186/s12937-023-00859-4)

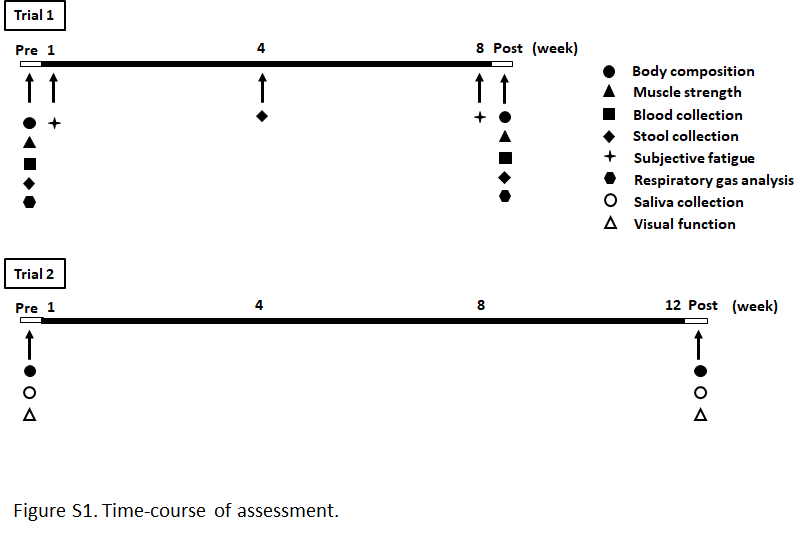

Supplement: Supplementary file 1 — Additional file 1: Figure S1. Time-course of assessment. [file 12937_2023_859_MOESM1_ESM.tif]

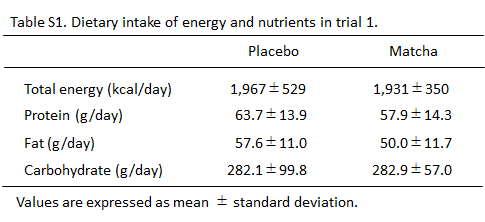

Supplement: Supplementary file 2 — Additional file 2: Table S1. Dietary intake of energy and nutrients in trial 1. [file 12937_2023_859_MOESM2_ESM.tif]

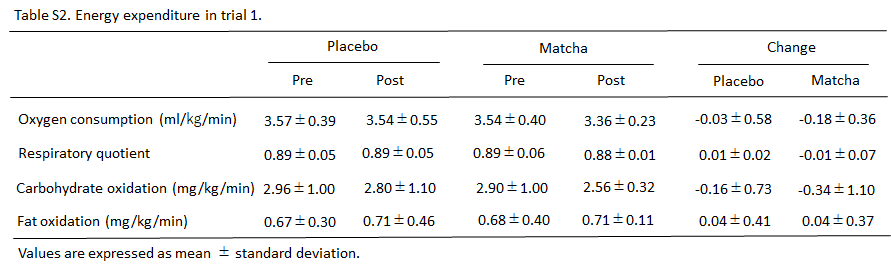

Supplement: Supplementary file 3 — Additional file 3: Table S2. Energy expenditure in trial 1. [file 12937_2023_859_MOESM3_ESM.tif]

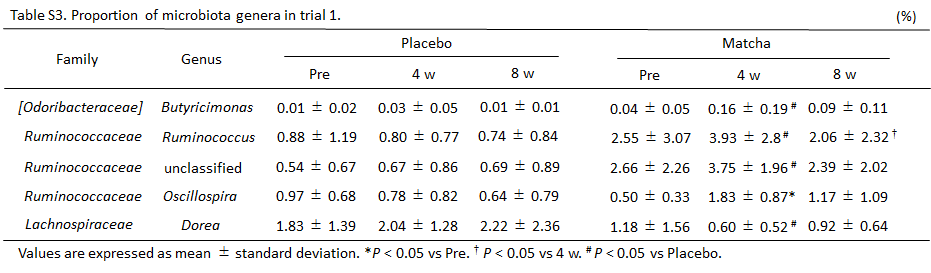

Supplement: Supplementary file 4 — Additional file 4: Table S3. Proportion of microbiota genera in trial 1. [file 12937_2023_859_MOESM4_ESM.tif]

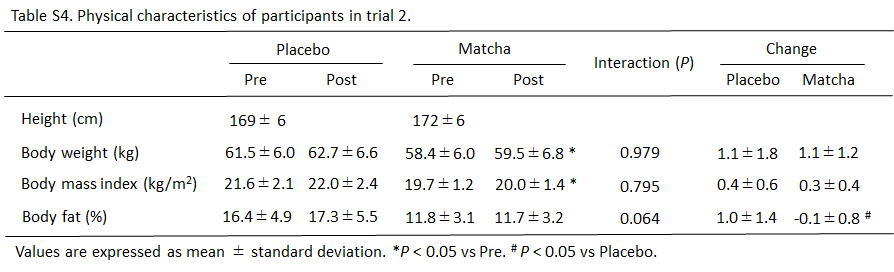

Supplement: Supplementary file 5 — Additional file 5: Table S4. Physical characteristics of participants in trial 2. [file 12937_2023_859_MOESM5_ESM.tif]

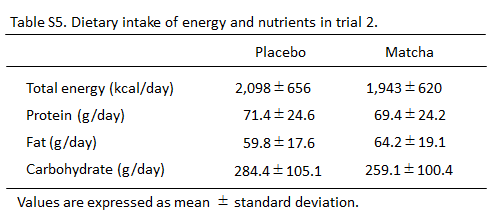

Supplement: Supplementary file 6 — Additional file 6: Table S5. Dietary intake of energy and nutrients in trial 2. [file 12937_2023_859_MOESM6_ESM.tif]
